# Crystal structure of tetrakis­(acetyl­acetonato)di­chloridodi-μ_3_-methano­lato-tetra-μ_2_-methano­lato-tetra­iron(III)

**DOI:** 10.1107/S2056989015013535

**Published:** 2015-07-29

**Authors:** Casseday P. Richers, Jeffery A. Bertke, Danielle L. Gray, Thomas B. Rauchfuss

**Affiliations:** a School of Chemical Sciences, University of Illinois at Urbana-Champaign, Urbana, Illinois 61801, USA

**Keywords:** crystal structure, cluster, iron(III), acetyl­acetonate, ouble cubane

## Abstract

The title complex, [Fe_4_(C_5_H_7_O_2_)_4_(CH_3_O)_6_Cl_2_] or [Fe_4_(acac)_4_(μ_2_-OMe)_4_(μ_3_-OMe)_2_Cl_2_] (acac = acetyl­acetonate), crystallizes in the ortho­rhom­bic *Pbca* space group with one half of the mol­ecule per asymmetric unit, the other half being completed by inversion symmetry. The core structure consists of a face-sharing double pseudo-cubane entity with two opposite corners missing. Weak C—H⋯Cl inter­molecular inter­actions result in a two-dimensional layered structure parallel to the *ac* plane.

## Abstract 2

The mol­ecular structure of [Fe_4_Cl_2_(acac)_4_(OMe)_6_] (acac = acetyl­acetonate) consists of a face-sharing double cubane cluster with two opposite corners missing. Weak C—H⋯Cl inter­molecular inter­actions result in a two-dimensional extended sheet structure normal to the *b* axis.

## Chemical context   

Metal silanolate complexes bearing meth­oxy and eth­oxy groups on silicon are relatively rare (Dupuy *et al.*, 2012 ▸) in comparison to *tert*-but­oxy­silanolate complexes (McMullen *et al.*, 1989 ▸, 1990 ▸; Nozaki *et al.*, 2002 ▸; Terry *et al.*, 1993 ▸, 1996 ▸; Truscott *et al.*, 2013 ▸). Nevertheless, such compounds may play a pivotal role in sol-gel reactions and in metal-catalysed curing reactions, such as room-temperature vulcanization (Cervantes *et al.*, 2012 ▸; Levitsky *et al.*, 2007 ▸; van Der Weij, 1980 ▸). 
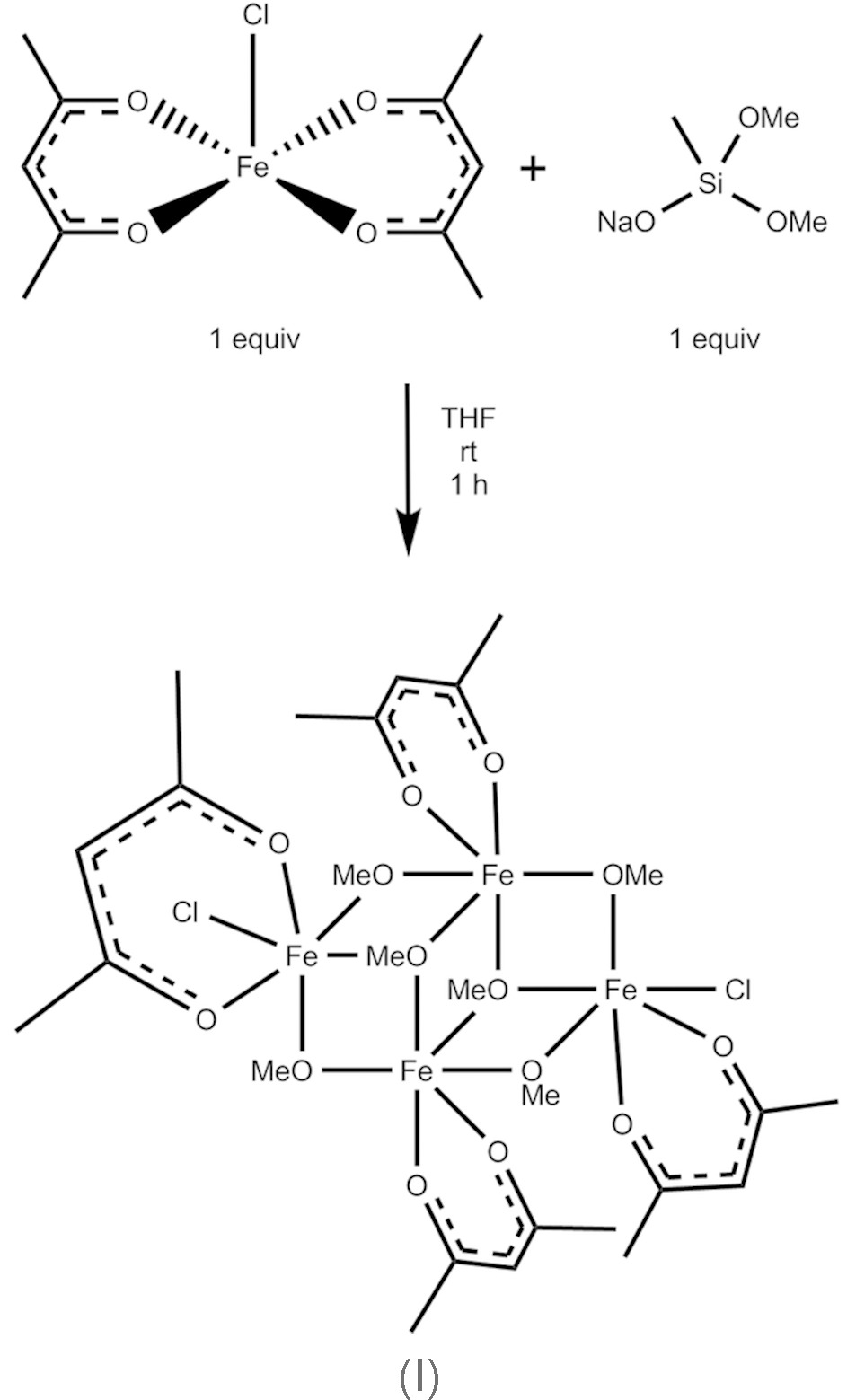


We have investigated the syntheses of metal meth­oxy­silanolates *via* the additions of NaOSi(OMe)_2_Me to metal halides and discovered that, in certain cases, the addition of NaOSi(OMe)_2_Me to a metal halide results in the formation of a methano­late complex instead of silanolate complex. In line with this observation, we now report that the addition of NaOSi(OMe)_2_Me to Fe(acac)_2_Cl results in the formation of a tetra­nuclear iron(III) methano­late compound, Fe_4_(acac)_4_(μ_2_-OMe)_4_(μ_3_-OMe)_2_Cl_2_, (I).

## Structural commentary   

The structure of (I) contains two crystallographically independent Fe^III^ metal atoms. Both cations are in approximately octa­hedral coordination environments. The coordination sphere of Fe1 is filled by the O atoms of one κ^2^-acac ligand [Fe1—O1 = 1.9971 (13) Å and Fe1—O2 = 1.9934 (13) Å], two μ_2_-methano­late groups [Fe1—O3 = 1.9861 (12) Å and Fe1—O5^i^ = 1.9885 (12) Å; symmetry code: (i) −*x* + 1, −*y* + 1, −*z* + 1], one μ_3_-methano­late group [Fe1—O4 = 2.2135 (12) Å], and one terminal chloride ligand [Fe1—Cl1 = 2.2776 (5) Å]. The coordination sphere of Fe2 is filled by the O atoms of one κ^2^-acac ligand [Fe2—O6 = 1.9717 (13) Å and Fe2—O7 = 1.9692 (12) Å], two μ_2_-methano­late groups [Fe2—O3 = 1.9755 (12) Å and Fe2—O5 = 1.9823 (12) Å], and two μ_3_-methano­late groups [Fe2—O4 = 2.0815 (12) Å and Fe2—O4^i^ = 2.0809 (12) Å]. The angles around both Fe1 and Fe2 distort significantly from the ideal values of 90 and 180° of a perfect octa­hedron. For Fe1, the *cis* angles range from 75.69 (5) to 98.40 (4)°, while the *trans* angles range from 164.47 (5) to 170.40 (3)°. The angles around Fe2 have narrower ranges, with *cis* being 78.95 (5)–96.48 (5)° and *trans* being 170.08 (5)–170.16 (5)°.

**Figure 1:**
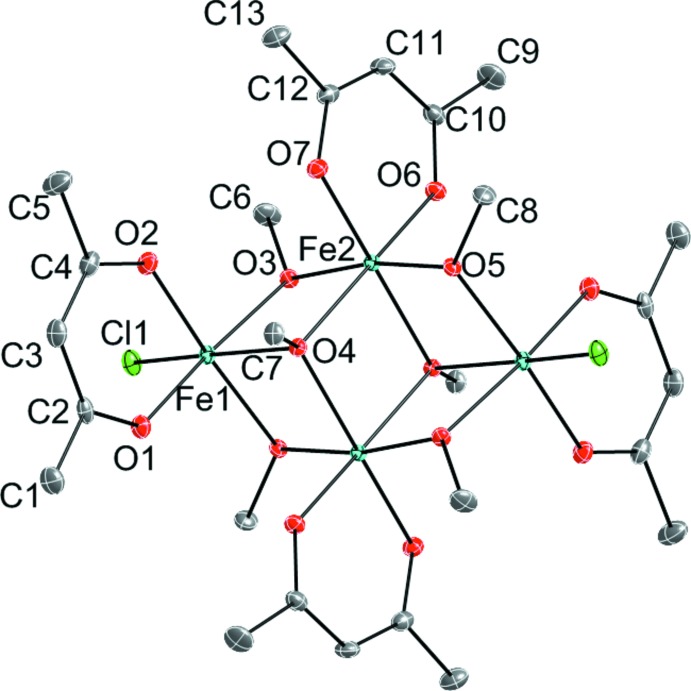
View of the molecular structure of (I), showing the atomic numbering and 35% probability displacement ellipsoids for the non-H atoms. The unlabeled atoms are related to the labeled ones by the symmetry operator (−*x* + 1, −*y* + 1, −*z* + 1). H atoms have been removed for clarity.

The mol­ecular structure of (I) (Fig. 1 ▸) can be described as an [Fe_4_(OMe)_6_] face-sharing double pseudo-cubane entity with two opposite corners missing. The outside of the cluster is decorated by one acac ligand per metal and the Fe atoms at either end of the cluster are coordinated by one chloride ion. Neighboring Fe⋯Fe distances range from 3.1997 (4) to 3.2175 (6) Å, while the Fe1⋯Fe1^i^ distance is 5.5702 (6) Å.

## Supra­molecular features   

**Figure 2:**
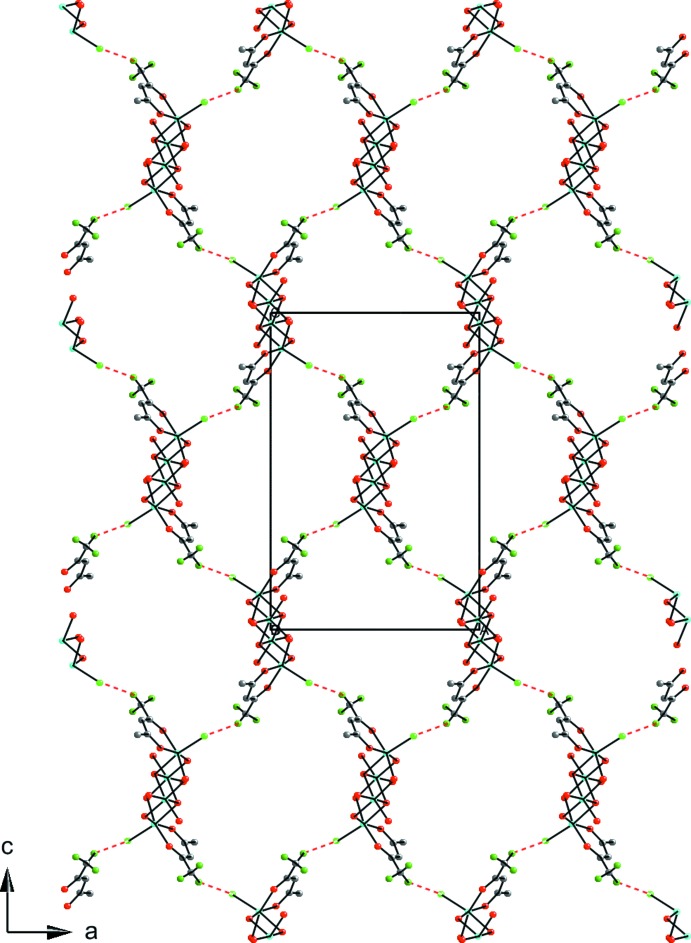
A view along the *b* axis of the extended two-dimensional network of (I) with an overlay of the unit cell. The inter­molecular Cl—H inter­ations are shown as dashed red lines. All C atoms except those in the hydrogen-bonded acac ligand and all H atoms except those of the hydrogen-bonded methyl group have been removed for clarity. Color key: blue = Fe, light-green = Cl, red = O, gray = C, and green = H.

There are no significant supra­molecular features to discuss with the extended structure of (I). There are weak inter­actions between the Cl^−^ ion and an acac ligand on neighboring mol­ecules (Table 1 ▸). Taking into account these weak inter­actions, the extended structure becomes layers of two-dimensional 4^4^-nets normal to the *b* axis (Fig. 2 ▸).

## Database survey   

One closely related complex, [Fe_4_(acac)_4_(OMe)_6_(N_3_)_2_], has previously been reported (Li *et al.*, 1997 ▸) in which N_3_^−^ takes the position of Cl^−^ in (I). The mol­ecular structure of the azide complex is very similar to that of (I), and can be described as the same [Fe_4_(OMe)_6_] face-sharing double cubane cluster with two opposite corners missing. The average Fe—O_acac_ distance of 1.978 Å is quite close to the average Fe—O_acac_ distance of 1.982 Å in (I). The average Fe—OMe distances in the azide complex (μ_2_-OMe: 1.977 Å; μ_3_-OMe: 2.124 Å) are also comparable to those in (I) (μ_2_-OMe: 1.983 Å; μ_3_-OMe: 2.125 Å).

A search of the Cambridge Structural Database (Groom & Allen, 2014 ▸) returned 14 complexes with an [Fe_4_(O*R*)_6_] cluster core similar to (I) (Abu-Nawwas *et al.*, 2009 ▸; Mulyana *et al.*, 2009 ▸). All of these materials, except the azide compound described above, use more complex, multidentate ligands to form the polynuclear entity. The [Fe_4_(O*R*)_6_] motif is present is 63 additional materials as part of a higher-order cluster complex (Ferguson *et al.*, 2013 ▸; Murugesu *et al.*, 2004 ▸).

## Synthesis and crystallization   

A solution of NaOSi(OMe)_2_Me (57 mg, 3.96 × 10 ^−4^ mol, 1 equivalent) in THF (3 ml) was added to a solution of Fe(acac)_2_Cl (200 mg, 3.96 × 10 ^−4^ mol, 1 equivalent) in THF (see Scheme). The mixture was stirred rapidly at room temperature, and a slight color change from a dark-red to a lighter red was observed. Removal of the solvent under vacuum resulted in the precipitation of an orange solid, which upon washing with dry Et_2_O (2 × 10 ml) left a yellow solid. The yellow solid was extracted into dry CH_2_Cl_2_ and filtered through Celite. The CH_2_Cl_­2_ was then removed under vacuum, leaving a yellow solid (54 mg, 6.16 × 10 ^−5^ mol, 62% yield). Crystals suitable for X-ray diffraction were grown by slow diffusion of pentane into a CH_2_Cl_2_ solution of the yellow solid.

## Refinement   

Crystal data, data collection and structure refinement details are summarized in Table 2 ▸. Methyl-H atom positions, *R*CH_3_, were optimized by rotation about *R*—C bonds, with idealized C—H, *R*—H and H⋯H distances (C—H = 0.98 Å). The remaining H atoms were included as riding idealized contributors (C—H = 0.95 Å). H atoms were assigned *U*_iso_(H) = 1.5*U*_eq_(C) for methyl H atoms and *U*_iso_(H) = 1.2*U*_eq_(C) otherwise. The 102 reflection was omitted from the final refinement because it was partially obscured by the shadow of the beam stop.

## Supplementary Material

Crystal structure: contains datablock(s) I. DOI: 10.1107/S2056989015013535/cv5491sup1.cif

Structure factors: contains datablock(s) I. DOI: 10.1107/S2056989015013535/cv5491Isup2.hkl

CCDC reference: 1412851

Additional supporting information:  crystallographic information; 3D view; checkCIF report

## Figures and Tables

**Table 1:** Hydrogen-bond geometry (, )

*D*H*A*	*D*H	H*A*	*D* *A*	*D*H*A*
C3H3Cl1^i^	0.95	2.91	3.797(2)	155
C5H5*B*Cl1^i^	0.98	2.91	3.800(2)	152

Symmetry code: (i) 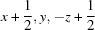.

**Table 2:** Experimental details

Crystal data	
Chemical formula	[Fe_4_(C_5_H_7_O_2_)_4_(CH_3_O)_6_Cl_2_]
*M*_r_	876.93
Crystal system, space group	Orthorhombic, *P* *b* *c* *a*
Temperature (K)	102
*a*, *b*, *c* ()	14.0714(6), 12.1888(4), 21.3543(7)
*V* (^3^)	3662.6(2)
*Z*	4
Radiation type	Mo *K*
(mm^1^)	1.76
Crystal size (mm)	0.38 0.37 0.23

Data collection	
Diffractometer	Bruker D8 Venture/Photon 100
Absorption correction	Integration (*SADABS*; Bruker, 2012 ▸)
*T*_min_, *T*_max_	0.568, 0.718
No. of measured, independent and observed [*I* > 2(*I*)] reflections	46682, 4559, 3837
*R*_int_	0.060
(sin /)_max_ (^1^)	0.668

Refinement	
*R*[*F* ^2^ > 2(*F* ^2^)], *wR*(*F* ^2^), *S*	0.028, 0.070, 1.04
No. of reflections	4559
No. of parameters	215
H-atom treatment	H-atom parameters constrained
_max_, _min_ (e ^3^)	0.39, 0.34

Computer programs: *APEX2*, *SAINT*, *XPREP* and *XCIF* (Bruker, 2013 ▸), *SHELXT* (Sheldrick, 2015*a* ▸), *SHELXL2013* (Sheldrick, 2015*b* ▸), *SHELXTL* (Sheldrick, 2008 ▸), *CrystalMaker* (CrystalMaker, 2014 ▸) and *publCIF* (Westrip, 2010 ▸).

## supplementary crystallographic information

### Crystal data

**Table:**

[Fe_4_(C_5_H_7_O_2_)_4_(CH_3_O)_6_Cl_2_]	*D*_x_ = 1.590 Mg m^−^^3^
*M**_r_* = 876.93	Mo *K*α radiation, λ = 0.71073 Å
Orthorhombic, *P**b**c**a*	Cell parameters from 9882 reflections
*a* = 14.0714 (6) Å	θ = 2.4–28.3°
*b* = 12.1888 (4) Å	µ = 1.76 mm^−^^1^
*c* = 21.3543 (7) Å	*T* = 102 K
*V* = 3662.6 (2) Å^3^	Prism, orange
*Z* = 4	0.38 × 0.37 × 0.23 mm
*F*(000) = 1808	

### Data collection

**Table:**

Bruker D8 Venture/Photon 100 diffractometer	4559 independent reflections
Radiation source: microfocus sealed tube	3837 reflections with *I* > 2σ(*I*)
Multilayer mirrors monochromator	*R*_int_ = 0.060
profile data from φ and ω scans	θ_max_ = 28.3°, θ_min_ = 2.9°
Absorption correction: integration (*SADABS*; Bruker, 2012)	*h* = −18→18
*T*_min_ = 0.568, *T*_max_ = 0.718	*k* = −15→16
46682 measured reflections	*l* = −28→28

### Refinement

**Table:**

Refinement on *F*^2^	0 restraints
Least-squares matrix: full	Hydrogen site location: inferred from neighbouring sites
*R*[*F*^2^ > 2σ(*F*^2^)] = 0.028	H-atom parameters constrained
*wR*(*F*^2^) = 0.070	*w* = 1/[σ^2^(*F*_o_^2^) + (0.0352*P*)^2^ + 1.8648*P*] where *P* = (*F*_o_^2^ + 2*F*_c_^2^)/3
*S* = 1.04	(Δ/σ)_max_ = 0.001
4559 reflections	Δρ_max_ = 0.39 e Å^−^^3^
215 parameters	Δρ_min_ = −0.34 e Å^−^^3^

### Special details

**Table:**

Experimental. One distinct cell was identified using *APEX2* (Bruker, 2013). Four frame series were integrated and filtered for statistical outliers using *SAINT* (Bruker, 2013) then corrected for absorption by integration using *SADABS* v2012/1 (Bruker, 2012). No decay correction was applied.
Geometry. All e.s.d.'s (except the e.s.d. in the dihedral angle between two l.s. planes) are estimated using the full covariance matrix. The cell e.s.d.'s are taken into account individually in the estimation of e.s.d.'s in distances, angles and torsion angles; correlations between e.s.d.'s in cell parameters are only used when they are defined by crystal symmetry. An approximate (isotropic) treatment of cell e.s.d.'s is used for estimating e.s.d.'s involving l.s. planes.
Refinement. Structure was phased by intrinsic phasing methods (XT, Sheldrick, 2013). Systematic conditions suggested the unambiguous space group. The space group choice was confirmed by successful convergence of the full-matrix least-squares refinement on *F*^2^. The final difference Fourier had no significant features. A final analysis of variance between observed and calculated structure factors showed little dependence on amplitude or resolution.

### Fractional atomic coordinates and isotropic or equivalent isotropic displacement parameters (Å^2^)

**Table:**

	*x*	*y*	*z*	*U*_iso_*/*U*_eq_	
Fe1	0.44600 (2)	0.60052 (2)	0.38841 (2)	0.01161 (7)	
Fe2	0.49483 (2)	0.38287 (2)	0.46636 (2)	0.01051 (7)	
Cl1	0.31634 (3)	0.65927 (4)	0.33408 (2)	0.01952 (11)	
O1	0.52694 (10)	0.73202 (11)	0.37204 (6)	0.0177 (3)	
O2	0.51458 (9)	0.52865 (11)	0.31778 (6)	0.0170 (3)	
O3	0.39890 (9)	0.45244 (10)	0.41172 (5)	0.0126 (2)	
O4	0.55491 (8)	0.53740 (10)	0.45395 (5)	0.0106 (2)	
O5	0.59118 (9)	0.33767 (10)	0.52897 (5)	0.0130 (3)	
O6	0.41693 (9)	0.25001 (10)	0.47819 (6)	0.0158 (3)	
O7	0.56371 (9)	0.31011 (10)	0.39767 (6)	0.0153 (3)	
C1	0.63600 (17)	0.85947 (17)	0.33019 (11)	0.0306 (5)	
H1A	0.5958	0.9116	0.3530	0.046*	
H1B	0.6452	0.8854	0.2872	0.046*	
H1C	0.6978	0.8535	0.3511	0.046*	
C2	0.58856 (14)	0.74858 (16)	0.32912 (9)	0.0190 (4)	
C3	0.61526 (14)	0.67189 (17)	0.28427 (9)	0.0204 (4)	
H3	0.6604	0.6933	0.2535	0.024*	
C4	0.57959 (13)	0.56530 (16)	0.28188 (8)	0.0169 (4)	
C5	0.61965 (15)	0.48447 (18)	0.23600 (9)	0.0246 (4)	
H5A	0.6607	0.4323	0.2581	0.037*	
H5B	0.6568	0.5236	0.2042	0.037*	
H5C	0.5676	0.4447	0.2157	0.037*	
C6	0.34499 (14)	0.38938 (16)	0.36761 (9)	0.0201 (4)	
H6A	0.3847	0.3723	0.3312	0.030*	
H6B	0.2894	0.4317	0.3541	0.030*	
H6C	0.3240	0.3210	0.3874	0.030*	
C7	0.65434 (12)	0.54563 (16)	0.43948 (8)	0.0151 (4)	
H7A	0.6918	0.5215	0.4756	0.023*	
H7B	0.6701	0.6220	0.4296	0.023*	
H7C	0.6690	0.4990	0.4034	0.023*	
C8	0.63734 (16)	0.23375 (17)	0.52457 (10)	0.0242 (5)	
H8A	0.5895	0.1754	0.5233	0.036*	
H8B	0.6785	0.2232	0.5611	0.036*	
H8C	0.6757	0.2313	0.4863	0.036*	
C9	0.35287 (16)	0.07273 (17)	0.46844 (10)	0.0258 (5)	
H9A	0.3624	0.0571	0.5130	0.039*	
H9B	0.3634	0.0057	0.4440	0.039*	
H9C	0.2878	0.0987	0.4617	0.039*	
C10	0.42174 (14)	0.15947 (15)	0.44786 (9)	0.0171 (4)	
C11	0.48468 (15)	0.13917 (15)	0.39877 (9)	0.0198 (4)	
H11	0.4823	0.0689	0.3796	0.024*	
C12	0.55074 (14)	0.21388 (16)	0.37582 (9)	0.0175 (4)	
C13	0.61370 (17)	0.18240 (18)	0.32192 (10)	0.0286 (5)	
H13A	0.6064	0.2360	0.2881	0.043*	
H13B	0.5957	0.1095	0.3067	0.043*	
H13C	0.6801	0.1812	0.3358	0.043*	

### Atomic displacement parameters (Å^2^)

**Table:**

	*U*^11^	*U*^22^	*U*^33^	*U*^12^	*U*^13^	*U*^23^
Fe1	0.01076 (13)	0.01374 (13)	0.01034 (12)	−0.00004 (9)	0.00102 (9)	0.00161 (9)
Fe2	0.01094 (13)	0.01008 (12)	0.01050 (13)	−0.00020 (9)	0.00027 (9)	−0.00061 (9)
Cl1	0.0182 (2)	0.0259 (2)	0.0145 (2)	0.00438 (18)	−0.00364 (17)	0.00256 (17)
O1	0.0174 (7)	0.0180 (7)	0.0179 (7)	−0.0018 (5)	0.0040 (5)	0.0027 (5)
O2	0.0156 (7)	0.0208 (7)	0.0146 (6)	0.0001 (5)	0.0040 (5)	0.0015 (5)
O3	0.0119 (6)	0.0147 (6)	0.0112 (6)	−0.0025 (5)	−0.0017 (5)	−0.0005 (5)
O4	0.0074 (6)	0.0125 (6)	0.0121 (6)	−0.0004 (5)	0.0015 (4)	0.0004 (5)
O5	0.0127 (6)	0.0126 (6)	0.0138 (6)	0.0031 (5)	−0.0001 (5)	−0.0005 (5)
O6	0.0164 (7)	0.0139 (6)	0.0171 (6)	−0.0030 (5)	0.0016 (5)	0.0001 (5)
O7	0.0166 (7)	0.0144 (6)	0.0149 (6)	−0.0002 (5)	0.0029 (5)	−0.0029 (5)
C1	0.0292 (12)	0.0239 (11)	0.0388 (13)	−0.0074 (9)	0.0114 (10)	0.0021 (9)
C2	0.0147 (9)	0.0205 (10)	0.0216 (10)	−0.0003 (8)	−0.0005 (8)	0.0080 (8)
C3	0.0154 (10)	0.0280 (10)	0.0177 (9)	−0.0016 (8)	0.0047 (7)	0.0064 (8)
C4	0.0134 (9)	0.0268 (10)	0.0105 (8)	0.0042 (8)	−0.0008 (7)	0.0044 (7)
C5	0.0230 (11)	0.0302 (11)	0.0205 (10)	0.0037 (9)	0.0077 (8)	−0.0007 (8)
C6	0.0198 (10)	0.0216 (10)	0.0191 (9)	−0.0059 (8)	−0.0080 (8)	−0.0011 (7)
C7	0.0076 (8)	0.0205 (9)	0.0172 (9)	−0.0002 (7)	0.0020 (7)	0.0018 (7)
C8	0.0272 (11)	0.0190 (10)	0.0263 (11)	0.0127 (8)	−0.0064 (9)	−0.0051 (8)
C9	0.0244 (11)	0.0176 (10)	0.0353 (12)	−0.0064 (8)	0.0005 (9)	0.0005 (8)
C10	0.0164 (9)	0.0131 (9)	0.0217 (9)	−0.0005 (7)	−0.0053 (7)	0.0014 (7)
C11	0.0236 (11)	0.0129 (9)	0.0229 (10)	−0.0008 (7)	−0.0019 (8)	−0.0056 (7)
C12	0.0193 (10)	0.0182 (9)	0.0151 (9)	0.0044 (8)	−0.0013 (7)	−0.0035 (7)
C13	0.0334 (13)	0.0252 (11)	0.0274 (11)	0.0016 (9)	0.0113 (9)	−0.0107 (9)

### Geometric parameters (Å, º)

**Table:**

Fe1—O3	1.9861 (12)	C3—C4	1.394 (3)
Fe1—O5^i^	1.9885 (12)	C3—H3	0.9500
Fe1—O2	1.9934 (13)	C4—C5	1.499 (3)
Fe1—O1	1.9971 (13)	C5—H5A	0.9800
Fe1—O4	2.2135 (12)	C5—H5B	0.9800
Fe1—Cl1	2.2776 (5)	C5—H5C	0.9800
Fe2—O7	1.9692 (12)	C6—H6A	0.9800
Fe2—O6	1.9717 (13)	C6—H6B	0.9800
Fe2—O3	1.9755 (12)	C6—H6C	0.9800
Fe2—O5	1.9823 (12)	C7—H7A	0.9800
Fe2—O4^i^	2.0809 (12)	C7—H7B	0.9800
Fe2—O4	2.0815 (12)	C7—H7C	0.9800
O1—C2	1.278 (2)	C8—H8A	0.9800
O2—C4	1.274 (2)	C8—H8B	0.9800
O3—C6	1.433 (2)	C8—H8C	0.9800
O4—C7	1.436 (2)	C9—C10	1.500 (3)
O4—Fe2^i^	2.0808 (12)	C9—H9A	0.9800
O5—C8	1.426 (2)	C9—H9B	0.9800
O5—Fe1^i^	1.9885 (12)	C9—H9C	0.9800
O6—C10	1.281 (2)	C10—C11	1.394 (3)
O7—C12	1.275 (2)	C11—C12	1.391 (3)
C1—C2	1.508 (3)	C11—H11	0.9500
C1—H1A	0.9800	C12—C13	1.502 (3)
C1—H1B	0.9800	C13—H13A	0.9800
C1—H1C	0.9800	C13—H13B	0.9800
C2—C3	1.390 (3)	C13—H13C	0.9800
			
O3—Fe1—O5^i^	91.96 (5)	O1—C2—C1	115.54 (18)
O3—Fe1—O2	87.24 (5)	C3—C2—C1	119.58 (18)
O5^i^—Fe1—O2	164.84 (5)	C2—C3—C4	123.69 (17)
O3—Fe1—O1	164.47 (5)	C2—C3—H3	118.2
O5^i^—Fe1—O1	90.08 (5)	C4—C3—H3	118.2
O2—Fe1—O1	86.80 (5)	O2—C4—C3	124.28 (18)
O3—Fe1—O4	75.92 (5)	O2—C4—C5	115.63 (18)
O5^i^—Fe1—O4	75.69 (5)	C3—C4—C5	120.07 (17)
O2—Fe1—O4	89.45 (5)	C4—C5—H5A	109.5
O1—Fe1—O4	89.70 (5)	C4—C5—H5B	109.5
O3—Fe1—Cl1	98.40 (4)	H5A—C5—H5B	109.5
O5^i^—Fe1—Cl1	97.01 (4)	C4—C5—H5C	109.5
O2—Fe1—Cl1	98.08 (4)	H5A—C5—H5C	109.5
O1—Fe1—Cl1	96.63 (4)	H5B—C5—H5C	109.5
O4—Fe1—Cl1	170.40 (3)	O3—C6—H6A	109.5
O7—Fe2—O6	89.95 (5)	O3—C6—H6B	109.5
O7—Fe2—O3	95.14 (5)	H6A—C6—H6B	109.5
O6—Fe2—O3	92.77 (5)	O3—C6—H6C	109.5
O7—Fe2—O5	92.33 (5)	H6A—C6—H6C	109.5
O6—Fe2—O5	93.76 (5)	H6B—C6—H6C	109.5
O3—Fe2—O5	170.08 (5)	O4—C7—H7A	109.5
O7—Fe2—O4^i^	170.08 (5)	O4—C7—H7B	109.5
O6—Fe2—O4^i^	95.27 (5)	H7A—C7—H7B	109.5
O3—Fe2—O4^i^	93.02 (5)	O4—C7—H7C	109.5
O5—Fe2—O4^i^	78.95 (5)	H7A—C7—H7C	109.5
O7—Fe2—O4	96.48 (5)	H7B—C7—H7C	109.5
O6—Fe2—O4	170.16 (5)	O5—C8—H8A	109.5
O3—Fe2—O4	79.28 (5)	O5—C8—H8B	109.5
O5—Fe2—O4	93.41 (5)	H8A—C8—H8B	109.5
O4^i^—Fe2—O4	79.52 (5)	O5—C8—H8C	109.5
C2—O1—Fe1	129.75 (13)	H8A—C8—H8C	109.5
C4—O2—Fe1	130.44 (13)	H8B—C8—H8C	109.5
C6—O3—Fe2	121.31 (11)	C10—C9—H9A	109.5
C6—O3—Fe1	119.96 (11)	C10—C9—H9B	109.5
Fe2—O3—Fe1	108.06 (6)	H9A—C9—H9B	109.5
C7—O4—Fe2^i^	118.09 (10)	C10—C9—H9C	109.5
C7—O4—Fe2	119.09 (10)	H9A—C9—H9C	109.5
Fe2^i^—O4—Fe2	100.48 (5)	H9B—C9—H9C	109.5
C7—O4—Fe1	120.95 (10)	O6—C10—C11	124.49 (18)
Fe2^i^—O4—Fe1	97.00 (5)	O6—C10—C9	115.14 (17)
Fe2—O4—Fe1	96.53 (5)	C11—C10—C9	120.37 (18)
C8—O5—Fe2	120.92 (11)	C12—C11—C10	124.99 (17)
C8—O5—Fe1^i^	120.97 (11)	C12—C11—H11	117.5
Fe2—O5—Fe1^i^	108.25 (6)	C10—C11—H11	117.5
C10—O6—Fe2	127.83 (12)	O7—C12—C11	124.67 (17)
C12—O7—Fe2	128.04 (12)	O7—C12—C13	115.52 (18)
C2—C1—H1A	109.5	C11—C12—C13	119.81 (17)
C2—C1—H1B	109.5	C12—C13—H13A	109.5
H1A—C1—H1B	109.5	C12—C13—H13B	109.5
C2—C1—H1C	109.5	H13A—C13—H13B	109.5
H1A—C1—H1C	109.5	C12—C13—H13C	109.5
H1B—C1—H1C	109.5	H13A—C13—H13C	109.5
O1—C2—C3	124.85 (18)	H13B—C13—H13C	109.5

Symmetry code: (i) −*x*+1, −*y*+1, −*z*+1.

### Hydrogen-bond geometry (Å, º)

**Table:**

*D*—H···*A*	*D*—H	H···*A*	*D*···*A*	*D*—H···*A*
C3—H3···Cl1^ii^	0.95	2.91	3.797 (2)	155
C5—H5*B*···Cl1^ii^	0.98	2.91	3.800 (2)	152

Symmetry code: (ii) *x*+1/2, *y*, −*z*+1/2.
